# Cutaneous leishmaniasis treatment and therapeutic outcomes in special populations: A collaborative retrospective study

**DOI:** 10.1371/journal.pntd.0011029

**Published:** 2023-01-23

**Authors:** Maria del Mar Castro, Joelle Rode, Paulo R. L. Machado, Alejandro Llanos-Cuentas, Marcia Hueb, Gláucia Cota, Isis Valentina Rojas, Yenifer Orobio, Oscar Oviedo Sarmiento, Ernesto Rojas, Juliana Quintero, Maria Inês Fernandes Pimentel, Jaime Soto, Carvel Suprien, Fiorela Alvarez, Ana Pilar Ramos, Rayssa Basílio dos Santos Arantes, Rosiana Estéfane da Silva, Claudia Marcela Arenas, Ivan Darío Vélez, Marcelo Rosandiski Lyra, Nancy Gore Saravia, Byron Arana, Neal Alexander

**Affiliations:** 1 Centro Internacional de Entrenamiento e Investigaciones Médicas (CIDEIM), Cali, Colombia; 2 Universidad Icesi, Cali, Colombia; 3 Drugs for Neglected Diseases *initiative* (DND*i*), Rio de Janeiro, Brazil; 4 Servico de Imunologia, Hospital Universitário Prof. Edgar Santos, Universidade Federal da Bahia, Salvador, Brazil; 5 Unidad de Leishmaniasis y Malaria, Instituto de Medicina Tropical Alexander von Humboldt, Universidad Peruana Cayetano Heredia, and Hospital Cayetano Heredia, Lima, Perú; 6 Universidade Federal de Mato Grosso, Hospital Universitário Júlio Müller (HUJM), Cuiabá, Mato Grosso, Brazil; 7 Instituto René Rachou, Fundação Oswaldo Cruz, Fiocruz, Belo Horizonte, Minas Gerais, Brazil; 8 Centro Dermatológico Federico Lleras Acosta E.S.E (CDFLA), Bogotá, Colombia; 9 Centro Universitario de Medicina Tropical–Universidad Mayor de San Simón (CUMT), Cochabamba, Bolivia; 10 PECET—Programa de Estudio y Control de Enfermedades Tropicales, Facultad de Medicina, Universidad de Antioquia, Medellín, Colombia; 11 Instituto Nacional de Infectologia Evandro Chagas (INI), Fundação Oswaldo Cruz, Rio de Janeiro, Brazil; 12 FUNDERMA (Fundación Nacional de Dermatología), Santa Cruz de la Sierra, Bolivia; 13 Drugs for Neglected Diseases *initiative* (DND*i*), Geneva, Switzerland; KU Leuven, BELGIUM

## Abstract

**Background:**

Treatment guidance for children and older adult patients affected by cutaneous leishmaniasis (CL) is unclear due to limited representation of these groups in clinical trials.

**Methods:**

We conducted a collaborative retrospective study to describe the effectiveness and safety of antileishmanial treatments in children ≤ 10 and adults ≥ 60 years of age, treated between 2014 and 2018 in ten CL referral centers in Latin America.

**Results:**

2,037 clinical records were assessed for eligibility. Of them, the main reason for non-inclusion was lack of data on treatment follow-up and therapeutic response (182/242, 75% of children and 179/468, 38% of adults). Data on 1,325 eligible CL patients (736 children and 589 older adults) were analyzed. In both age groups, disease presentation was mild, with a median number of lesions of one (IQR: 1–2) and median lesion diameter of less than 3 cm. Less than 50% of the patients had data for two or more follow-up visits post-treatment (being only 28% in pediatric patients). Systemic antimonials were the most common monotherapy regimen in both age groups (590/736, 80.2% of children and 308/589, 52.3% of older adults) with overall cure rates of 54.6% (95% CI: 50.5–58.6%) and 68.2% (95% CI: 62.6–73.4%), respectively. Other treatments used include miltefosine, amphotericin B, intralesional antimonials, and pentamidine. Adverse reactions related to the main treatment were experienced in 11.9% (86/722) of children versus 38.4% (206/537) of older adults. Most adverse reactions were of mild intensity.

**Conclusion:**

Our findings support the need for greater availability and use of alternatives to systemic antimonials, particularly local therapies, and development of strategies to improve patient follow-up across the region, with special attention to pediatric populations.

## Author summary

Cutaneous leishmaniasis (CL) is a vector-borne disease caused by a parasite called *Leishmania* that affects more than 1 million people worldwide each year and mostly causes chronic skin ulcers. The first line treatment continues to be systemic antimonials, despite drawbacks such as numerous daily injections and toxic side effects. Some groups, including children and older adults, are usually not included in clinical trials and, therefore, specific treatment guidance for them is uncertain due to the scarcity of robust evidence. RedeLEISH, a Latin American network of leishmaniasis researchers, conducted a retrospective study of the medical records of children ≤ 10 years of age and adults ≥ 60 years of age, treated between 2014 and 2018 in ten CL referral centers in the region, with the aim of learning about and documenting the effectiveness and tolerability of routine antileishmanial treatments, and ultimately contributing to improving the management of these patient groups. Our findings, based on 1,325 eligible patients’ records (589 adults ≥ 60 years of age and 736 children ≤ 10 years of age), showed that in both age groups the disease presents predominantly in a mild form, but that the most commonly used treatment, systemic antimonials, has limited effectiveness. This supports and encourages greater use of local therapies, and other treatment alternatives in these special populations. The results of this study also highlight the need to develop strategies for better follow-up of routine CL treatment and determination of effectiveness, as lack of such information was the principal criterion for exclusion of patients screened for eligibility, and less than 50% of eligible patients had information on two or more follow-up visits.

## Introduction

Cutaneous leishmaniasis (CL) is a parasitic disease caused by over 15 different species of the protozoan parasite *Leishmania*. The exact incidence of CL is not known, but it is estimated that more than 1 million new cases occur each year in approximately 90 countries worldwide[1]. In the Americas, CL is endemic in 18 countries and a total of 1,028,054 new cases were reported between 2001 and 2019. In 2020, 39,705 new cases were reported, with 81% concentrated in Brazil (16,432), Colombia (6,161), Peru (4,178), Nicaragua (3,443), and Bolivia (2,059). Of the new cases in 2020, 11.5% (4,560) were reported in children under 10 years of age, with this proportion being over 30% in some countries [2]; in 2019 approximately 15% of the cases occurred in adults aged 50 years or more [3]. Migration, demographic changes, deforestation, and uncontrolled urbanization have provoked an increase in the intra- and/or peri-domestic transmission of CL, which partly explains an increasing incidence in children and older adults [4,5].

Pentavalent antimonials continue to be the first-line treatment in many countries despite their toxicity, parenteral administration, and high cost. Other treatment options include miltefosine, the only efficacious oral treatment, and pentamidine isethionate, which is recommended as first line treatment for lesions caused by *L*. *guyanensis* in Brazil [6,7] and French Guyana [8,9]. Alternative or second line systemic treatments for special cases include amphotericin B deoxycholate or liposomal formulation, pentamidine isethionate, or ketoconazole [6]. Local interventions, such as intralesional antimonials and thermotherapy, are recommended by PAHO in the Americas for the treatment of CL involving localized single and small lesions of up to 3 cm diameter [6] that are not located on the face or articulations.

Vulnerable populations, such as children and older adults, have not usually been included in clinical trials for safety (e.g., contraindications due to the presence of comorbidities) and ethical considerations of potential risks. Clinical trials for CL treatments that have enrolled pediatric patients rarely report outcomes separately for children and, when available, they have revealed lower therapeutic response compared to adults [10]. Therefore, treatment guidance in these special populations is unclear due to the lack of robust evidence for effectiveness of the different treatment regimens. Consequently, case management is often based on clinical experience, case reports or small observational studies. Available information is scarce and scattered across different referral health facilities, hampering data access, pooling of data, and analysis of a large number of cases.

Seeking to address this gap, the RedeLEISH, a Latin American network of investigators and collaborators in leishmaniasis, proposed a collaborative project for retrospective data collection and sharing. The aim of this project is to describe the effectiveness and safety of antileishmanial treatments used in children ≤ 10 years of age and adults ≥ 60 years of age during a 5-year period (2014–2018) in ten CL referral centers in Latin America.

## Methods

### Ethics statement

The study protocol was approved by the institutional Ethics Committees of each of the ten participating institutions. No personal identifying information was shared as part of the study. A unique study number was assigned to each patient record. Waivers of informed consent were obtained for all sites because the retrospective request of informed consent was considered infeasible. Approval numbers and dates are listed for each site:

- Centro de Referência em Leishmaniose Posto de Saúde de Corte de Pedra (CSCP)/ C-HUPES, Universidade Federal da Bahia (UFBA), Salvador, Bahia, Brazil (approval date: 19/12/2019; CAAE 26607219.3.1001.5577)
- Instituto René Rachou (IRR), Fundação Oswaldo Cruz, FIOCRUZ Minas, Belo Horizonte, Minas Gerais, Brazil. (approval date: 13/02/2020; CAAE 26607219.3.2003.5091)
- Instituto Nacional de Infectologia Evandro Chagas (INI), Fundação Oswaldo Cruz, FIOCRUZ, Rio de Janeiro, Rio de Janeiro State, Brazil. (approval date: 16/06/2020; CAAE 26607219.3.2001.5262)
- Universidade Federal de Mato Grosso, Hospital Universitário Júlio Müller (HUJM), Cuiabá, Mato Grosso, Brazil. (approval date: 26/06/2020; CAAE 26607219.3.2002.5541)
- Centro Internacional de Entrenamiento e Investigaciones Médicas (CIDEIM), Cali, Colombia. (approval date: 08/07/2019; Number: 1293)
- Programa de Estudio y Control de Enfermedades Tropicales (PECET)–Universidad de Antioquia, Medellín, Colombia. (approval date: 28/08/2019)
- Centro Dermatológico Federico Lleras Acosta E.S.E (CDFLA), Bogotá, Colombia. (approval date: 12/12/2019; Number: 201902030032283)
- Centro Universitario de Medicina Tropical–Universidad Mayor de San Simón (CUMT), Cochabamba, Bolivia. (approval date: 27/08/2019)
- Hospital dermatológico de Jorochito /Fundación Nacional de Dermatologia (Funderma), Santa Cruz de la Siera, Bolivia. (approval date: 27/08/2019)
- Universidad Peruana Cayetano Heredia–Hospital Cayetano Heredia (UPCH), Lima, Peru. (approval date: 01/08/2019; Number: 085–019)

### Study design

We conducted a retrospective study based on data collected from the clinical records of pediatric patients of ≤ 10 years of age and adults of ≥ 60 years of age, treated for CL between January 2014 and December 2018. The study was conducted in ten reference centers for CL in Bolivia, Brazil, Colombia, and Peru. The current report is in accordance with the STROBE guidelines [11], S1 Table.

### Eligibility criteria

The study included patients of any gender and ethnicity, with age ≤10 or ≥ 60 years at the time of treatment for a parasitological or clinical and epidemiological diagnosis of CL. Patients were included independently of the treatment received in the participating institution and had at least one evaluation of therapeutic response at any time after completion of treatment. Patients presenting mucosal, diffuse, or disseminated forms of the disease were excluded, as well as those for whom information on the treatment regimen was unavailable.

### Outcome definitions

The following outcome definitions were used based on Olliaro *et al*. [12]:

- Initial cure: complete re-epithelialization of the lesion between D90 and D100 after the start of treatment.
- Final cure: complete re-epithelialization of the lesion between D180 and D360 after the start of treatment.
- Therapeutic failure: < 50% re-epithelization of the area of the lesion at any time between D42 and D63 after the start of treatment, *or* incomplete re-epithelialization of the lesion between D90 and D360 after the start of treatment.
- Relapse: reappearance of a nodule, plaque, or ulceration after cure [13].

### Data collection and management

An offline data collection tool in Microsoft Access, version 97–2003, was developed based on a digital form that was discussed and reviewed by the participating investigators. All participating sites were trained in the use of the database, and user manuals were developed to facilitate adequate management and data entry standardization at all sites.

Investigators at each participating institution identified eligible patients by searching their respective database (medical records, case report forms, previous studies, health surveillance case notification system). After patient screening, data collection and entry were performed at each site and sent regularly to CIDEIM, where they were gathered into a single database. This database comprised sociodemographic and clinical variables, as well as information on history of leishmaniasis, treatment, therapeutic response, and adverse drug reactions (adverse events related to the treatment). Information on therapeutic response was collected for up to two visits. When this information was available for more than two follow-up visits, the procedure established was to include those data nearest to D90 after the beginning of treatment. Data on relapse was collected for a period of up to 12 months after the beginning of the treatment received during the study period, when available.

Critical data were checked for completion and potential inconsistencies and queries were generated in a pre-defined format and issued to the participating sites.

Adverse drug reactions (a response to a drug which is noxious and unintended and which occurs at doses normally used in man for prophylaxis, diagnosis, or therapy of disease[14]) were collected and classified by System Organ Class (SOC) and standardized using nomenclature from the Common Terminology Criteria for Adverse Events (CTCAE) v5.0 of November 2017 [15]. Concomitant treatments were also manually classified by drug category, following the USP Therapeutic Categories Model Guidelines [16].

### Statistical analysis

Statistical analysis was performed in Stata/SE, version 15 according to a statistical analysis plan that was developed and agreed upon with the investigators. Categorical variables are presented as counts and proportions, with 95% confidence intervals. Continuous variables are presented as number of observations (*n*), mean and standard deviation (SD), or median and interquartile range (IQR).

## Results

**Fig 1:**
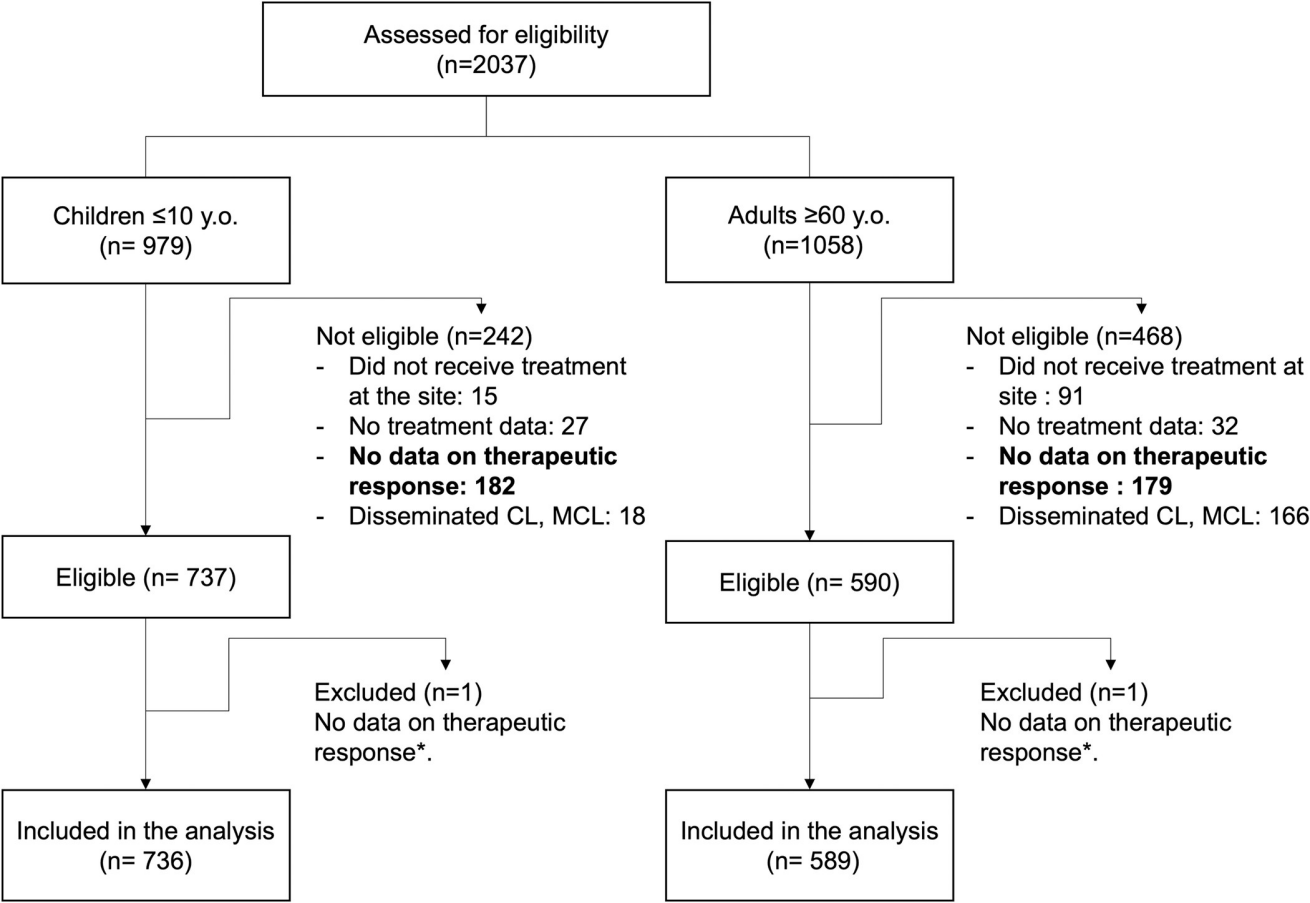
Screening and enrolment. * Excluded after the data cleaning and response to queries from the sites.

Clinical records of 2,037 patients were assessed for eligibility at the ten sites: 979 children ≤ 10 years of age and 1,058 adults aged ≥ 60 years. They were drawn from a total of 9,665 CL patients who sought care in the participating sites. Among screened records, the main reason for non-eligibility of both children and adults was lack of data on treatment follow-up and therapeutic response (182/242, 75% of children and 179/468, 38% of adults). In adults, this was closely followed by presentation of disseminated CL, mucosal, or mucocutaneous leishmaniasis (MCL) (35.4%, n = 166/468), Fig 1. Data on 1,325 eligible CL patients were collected in the ten study sites (59 from Bolivia, 873 from Brazil, 178 from Colombia, and 215 from Peru), S2 Table.

### Clinical and sociodemographic characteristics

In total, 736 (55.5%) study cases were children ≤ 10 years of age and 589 (44.5%) were adults ≥ 60 years of age. Most participants were male and of mixed-race ethnicity. The median age of the pediatric patients was 5 years (IQR: 3–7.5) and 68 years (IQR: 63–74) for the adults. Most patients presented with their first episode of CL (91.7% of children and 94.1% of adults) and did so after a short duration of disease. In children, the median time since symptom onset was 4 weeks (IQR: 3–8), while it was 8 weeks (IQR: 4–16) in adults (Table 1). Overall, disease presentation in the study population was mild, with a median number of lesions of one (IQR: 1–2) and the median size of lesion (defined as the largest diameter) was less than 30 mm for both study groups. Ulcerated lesions were the most frequent clinical presentation in both study groups. Lesions were smaller in the pediatric patients (median diameter 16 mm, IQR: 10–29) compared to the older adults (median diameter 25 mm, IQR: 13–40). Regarding anatomic location, head and neck were the most affected areas in children (n = 244, 33.5%), while the lower limbs were the most affected sites (n = 256, 43.7%) in older adults ≥ 60 years (Table 2).

**Table 1:** Clinical and demographic characteristics of patients.

	Patients ≤ 10 years		Patients ≥ 60 years	
	736		589	
***Demographic characteristics***				
Sex, female: n (%)	345	(46.9%)	251	(42.6%)
Age, years: median (IQR)	5	(3–7.5)	68	(63–74)
Ethnicity: n (%)				
African descent	28	(3.8%)	23	(3.9%)
White	8	(1.1%)	39	(6.6%)
Indigenous	15	(2%)	0	(0%)
Mixed ethnicity	197	(26.7%)	249	(42.3%)
No data	488	(66.3%)	278	(47.2%)
***Clinical characteristics***				
Weight. Mean (SD)	20.1	(7.9)	64	(14.2)
Type of case: n (%)				
New	673	(91.4%)	554	(94.1%)
Relapse	50	(6.8%)	14	(2.4%)
New lesion (with history of previous CL)	4	(0.5%)	18	(3.1%)
No data	9	(1%)	3	(0.5%)
Time from beginning of symptoms. Weeks. Median (IQR)	4	(3–8)	8	(4–16)
***Clinical antecedents***				
Concomitant diseases: n (%)				
Yes	19	(2.6%)	360	(61.1%)
No data	14	(1.9%)	15	(2.5%)
None, not applicable	703	(95.5%)	214	(36.3%)
Among patients with concomitant disease: n (%)	19	(2.6%)	360	(61.1%)
HIV	0	0%	2	(0.6%)
Diabetes	0	0%	89	(24.7%)
Hypertension	0	0%	269	(74.7%)
Hepatic disease	0	0%	5	(1.4%)
Cardiac disease	2	(10.5%)	41	(11.4%)
Kidney disease	0	0%	12	(3.3%)
Cancer	0	0%	8	(2.2%)
Other infection	16	(84.2%)	75	(20.8%)
Current treatments (taking any drug): n (%)				
Yes	14	(1.9%)	206	(35%)
No data	16	(2.2%)	132	(22.4%)
None, not applicable	706	(95.9%)	251	(42.6%)

IQR = interquartile range; SD = standard deviation; CL = cutaneous leishmaniasis

**Table 2:** Characteristics of cutaneous lesions.

Characteristics of cutaneous lesions	Patients ≤ 10 years		Patients ≥ 60 years	
Number of lesions. Median (IQR)	1	(1–2)	1	(1–2)
Number of lesions: n (%) *				
1	461	(63.2%)	378	(64.5%)
2	145	(19.9%)	105	(17.9%)
3	58	(8%)	48	(8.2%)
≥4	65	(8.9%)	55	(9.4%)
Anatomic site (based on number of cases) *. Cases with at least one lesion located on: n (%)				
Head/neck	244	(33.5%)	113	(19.3%)
Upper limbs	192	(26.3%)	180	(30.7%)
Lower limbs	236	(32.4%)	256	(43.7%)
Trunk	54	(7.4%)	37	(6.3%)
No data	3	(0.4%)	0	(0%)
***Total number of lesions***	***1222***		***1002***	
Anatomic site (based on number of lesions): n (%)				
Head/neck	412	(33.7%)	189	(18.9%)
Upper limbs	314	(25.7%)	310	(30.9%)
Lower limbs	391	(32%)	412	(41.1%)
Trunk	102	(8.3%)	89	(8.9%)
No data	3	(0.2%)	2	(0.2%)
Type of lesions: n (%)				
Ulcer	638	(87.5%)	515	(87.9%)
Other	89	(12.2%)	67	(11.4%)
No data	2	(0.3%)	4	(0.7%)
Lesion size. Diameter in mm of the largest lesion: Median (IQR)	16	(10–29)	25	(13–40)
Area of the largest lesion mm^2^. Median (IQR) **	659.7	(254.4–1649.3)	1451.4	(427.3–4146.9)
Ulcer size. Diameter in mm of the largest ulcer: Median (IQR)	9	(5–15)	15	(10–27)
Area of the largest ulcer mm^2^. Median (IQR) **	169.6	(78.5–490.1)	565.5	(219.9–1696.5)

* Number of patients with data of cutaneous lesions; **Area of lesion was calculated as an ellipse (*π · a · b)*. IQR = interquartile range

Sixty-one percent of patients ≥ 60 years of age had at least one concomitant disease, the most common being hypertension (74.7%) and diabetes (24.7%), and 35% reported the use of some concomitant medication. In contrast, a small proportion of children had comorbidities (2.6%) with other infections being the most common, and 1.9% reported using a concomitant medication (Table 1).

Among the patients ≥ 60 years of age, 74.7% (440/589) had parasitological confirmation of CL, mostly by direct smear (n = 320, 87.2% positive), followed by culture (n = 169, 69.8% positive) and polymerase chain reaction (PCR, n = 127, 95.3% positive). In 100 patients, other diagnostic approaches (immunohistochemistry, histopathology) were undertaken to confirm diagnosis. In contrast, only 36.5% (n = 269) of children had parasitological diagnosis. This disparity is due to the use of the leishmanin (Montenegro) skin test as a diagnostic tool in the site that contributed to the largest number of pediatric cases (CSCP), where all patients (n = 427) were recorded as having clinical-epidemiological diagnosis. This means that the definition of a CL case took into account the following: skin lesion(s), epidemiological factors for CL infection, and leishmanin test result (if done). Among pediatric patients with parasitological confirmation, direct smear was the most frequently employed method (n = 267, 92.1% positive), followed by histopathology (n = 61, 13.1% positive) and culture (n = 55, 70.9% positive). Only 19 cases were identified using other diagnostic methods (PCR or immunohistochemistry).

*Leishmania* species were identified in 107 (24.3%) adult patients, *L*. *(V*.*) braziliensis* being the most common species (n = 95, 88.8%), all treated in CSCP in Brazil, followed by *L*. *panamensis* (n = 11, 10.3%) and *L*. *infantum* (n = 1, 0.9%). Only 17 pediatric patients had species data (all from Colombia). Of them, 15 were infected with *L*. *(V*.*) panamensis*, one *L*. *mexicana*, and one *L*. *guyanensis* (S3 Table).

### Antileishmanial treatments and outcomes (cure and relapse)

#### Children

For monotherapy regimens, 80.2% of the patients were treated with systemic antimonials (n = 590), followed by miltefosine (n = 43, 5.8%) and intralesional (IL) antimonials (n = 16, 2.2%). Other monotherapies, including liposomal amphotericin B and pentamidine, were administered in 14 pediatric patients (1.9%). Thermotherapy was administered to only 2 patients. Seventy-three (9.9%) children received combination treatments, of whom 66 received systemic antimonials plus imiquimod.

**Fig 2:**
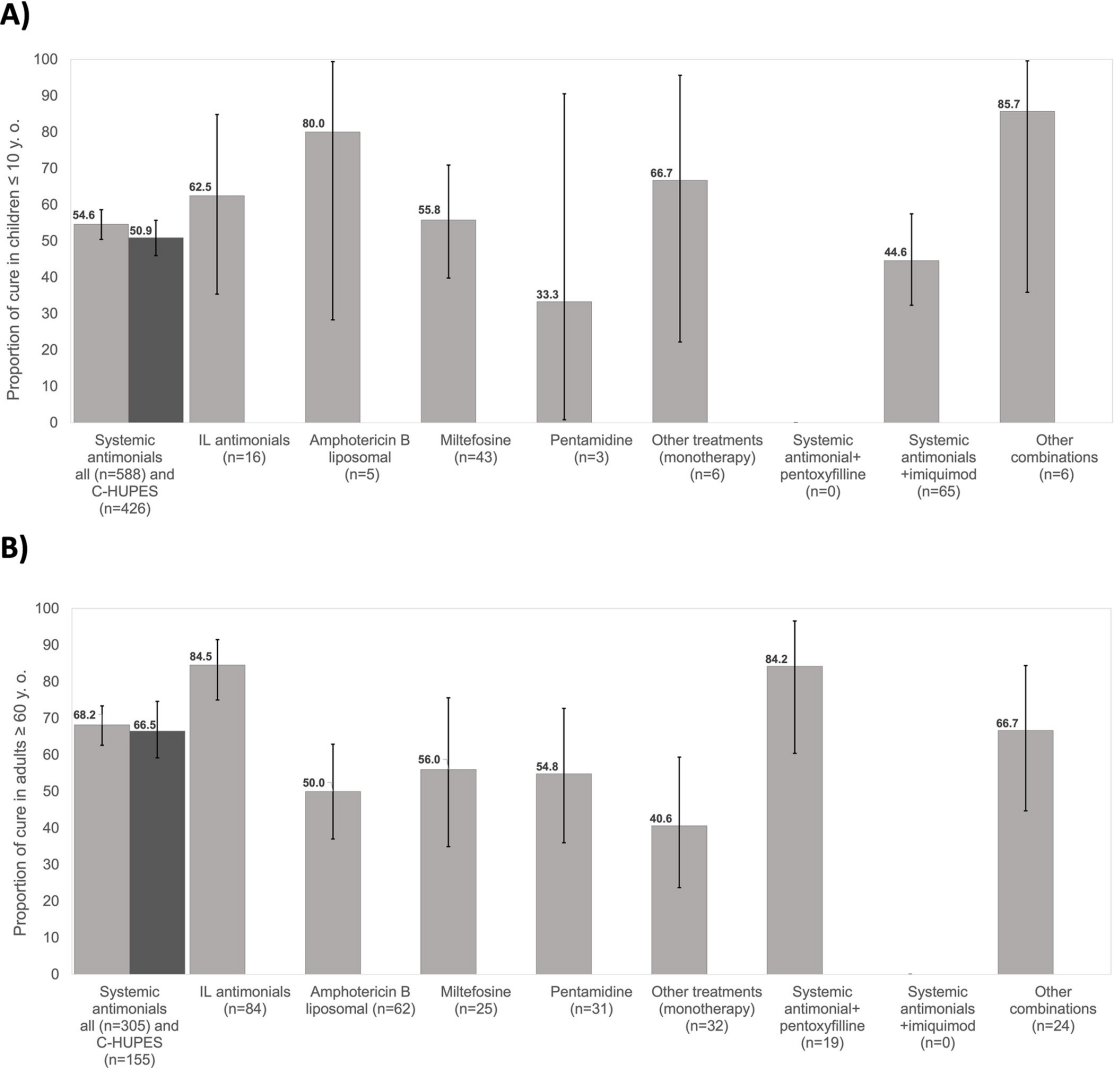
Overall cure rate A) children ≤ 10 years of age, B) adults ≥ 60 years of age. Detailed description of the outcomes and follow-up times is available in S4 and S5 Tables.

Initial cure rate (day 90–100) for systemic antimonials was 52% (n = 102/196, 95% CI: 44.8–59.2%). The overall cure rate, which corresponds to cumulative rates of cure defined at any moment of the follow-up (after end of treatment), ranged between 44.6% (95% CI: 32.6–57.4%) for imiquimod+antimonials combination, to 55.8% for miltefosine (95% CI: 39.8–70.9%). The overall cure rate for systemic antimonials was 54.6% (95% CI, 50.46–58.6%), Fig 2A. The number of cures for other treatment regimens with small numbers of treated patients include IL antimonials (10/16), amphotericin B (liposomal, 4/5), and pentamidine (1/3), Fig 2A.

A total of 345 children (46.9%) had data on relapse at any time of follow-up after treatment. Relapse occurred in 11.4% of pediatric patients treated with systemic antimonials (95% CI: 8.1–15.3%). Relapse estimates for other treatment options were not calculated due to the small number of patients with available data: 5 patients for miltefosine, 1 each for pentamidine and IL antimonials, and 12 for imiquimod+systemic antimonials (S4 Table).

#### Adults ≥ 60 years of age

Systemic antimonials were the most common monotherapy regimen in the study population, being administered to 308 (52.3%) patients. Liposomal amphotericin B was administered to 62 patients (10.5%), notably all from Brazil and 50 (82%) of them with concomitant diseases (S7 Table). Other regimens, such as miltefosine (n = 25, 4.2%) or pentamidine (n = 31, 5.3%) were used less frequently in this age group.

Notably, use of local therapies was limited, with only 84 (14.3%) of study patients receiving intralesional antimonials. Thermotherapy was administered to 19 patients. 43 patients (7.3%) received combination therapies and among them, the most frequent combination was systemic antimonial plus pentoxifylline (n = 19).

Initial cure rate (day 90–100) with systemic antimonials was 73.8% (95% CI: 60.9–84.2%). Overall cure (cumulative rates of cure defined at any moment of the follow-up after end of treatment) ranged between 50% (95% CI: 37.02–62.9%) for liposomal amphotericin B to 84.5% for IL antimonials (95% CI: 74.99–91.5%). Overall cure for systemic antimonials was 68.2% (95% CI: 62.6–73.39%), Fig 2B.

Data on relapse was available for 263 adult patients (44.7%) at any follow-up time after treatment. A relapse rate of 12.8% (95% CI: 8.2–18.7%) was estimated for systemic antimonials, and 6.3% for IL antimonials (95% CI: 0.77–20.8%). These were the only reliable estimates, since data on relapse for other treatment regimens was scarce (S5 Table).

### Treatment follow-up and compliance with treatment

#### Children

Based on exclusion criteria, all patients included in the study had at least one evaluation after the end of treatment; of them, 724 pediatric patients (98.4%) had information on the date of follow-up. However, only 28.1% patients had two or more follow-up visits post-treatment. The mean time to the first evaluation was 89.8 days (SD: 110.35), counted from the first day of treatment, and the mean time to the second evaluation was 155 days (SD: 165.8), Table 3.

**Table 3:** Treatment interruption per treatment regimen and age group.

Treatment interruption (due to adverse drug reactions or other reason). n/n treated (main treatment*) (%)	Patients ≤ 10 years		Patients ≥ 60 years	
Systemic antimonials	18/663	(2.7%)	43/335	(12.8%)
Intralesional antimonials	0/16	(0%)	4/87	(4.6%)
Amphotericin B deoxycholate	0/0	(0%)	1/1	(100%)
Liposomal amphotericin B	0/5	(0%)	10/62	(16.1%)
Miltefosine	8/43	(18.6%)	4/32	(12.5%)
Pentamidine	0/3	(0%)	1/31	(3.2%)
**Patients with at least one follow-up visit**	724	(98.4%)	583	(99%)
Time to the first follow-up in days. Mean (SD)	89.8	(110.35)	76.9	(53.37)
**Patients with two follow-up visits**	207	(28.1%)	287	(48.7%)
Time to the second follow-up in days. Mean (SD)	155	(165.80)	160.6	(112.44)
**Treatment interruption (including all treatments, due to adverse drug reactions or other reason): n (%)**				
Yes	26	(3.5%)	63	(10.7%)
No	702	(95.4%)	500	(84.9%)
No data	6	(0.8%)	12	(2%)
Not applicable	2	(0.3%)	14	(2.4%)
**In case of interruption, type: n (%)**				
Temporary	15	(57.7%)	26	(41.3%)
Definitive	5	(19.2%)	33	(52.4%)
No data	6	(23.1%)	4	(6.3%)
**In case of interruption, reason: n (%)**				
Adverse drug reaction	5	(19.2%)	36	(57.1%)
Other	3	(11.5%)	5	(7.9%)
No data	18	(69.2%)	22	(34.9%)

*Include all patients receiving systemic drugs (including in combination with other drugs)

Compliance with the dosing scheme of antileishmanial treatments was high for antimonials and amphotericin B, with more than 93.8% of pediatric patients receiving the recommended dose according to PAHO guidelines (S6 Table). Only 26 children (3.5%) interrupted treatment, 18 receiving systemic antimonials and 8 receiving miltefosine. Adverse drug reactions were the reason for treatment interruption in five of these patients, and five treatment interruptions were definitive (Table 3).

#### Adults ≥ 60 years of age

Consistent with exclusion criteria, all patients had at least one visit after the end of treatment (583; 99% of these with data on the date of follow-up). A higher proportion (48.7% vs 28.1%) of older adults than children had two or more follow-up visits. Mean time to the first visit was 76.9 days (SD: 53.37) and the mean time to the second follow-up visit was 160.6 days (SD: 112.4, Table 3).

Compliance with the dosing scheme of antileishmanial drugs was, again, high for antimonials (IL and systemic), with more than 93.1% of patients receiving the recommended dose according to PAHO guidelines (S6 Table). The proportion of patients receiving the recommended dose for miltefosine and amphotericin B was lower (78.1% and 69.4%, respectively).

Treatment interruptions occurred in 63 older adult patients (10.7%), mainly in those receiving systemic antimonials (12.8%) either as monotherapy or in combination, and liposomal amphotericin B (16.1%). Only 4 patients (4.6%) receiving intralesional antimonials experienced interruption of treatment, as did 4 of those receiving miltefosine (12.5%). Adverse drug reactions were the reason for treatment interruption in 36 patients (57.1%). Thirty-three treatment interruptions were definitive (Table 3).

### Adverse drug reactions

#### Children

A total of 86 pediatric patients (11.9%) presented adverse drug reactions (ADRs) to the main treatment regimens. The total number of such ADRs reported in the study was 174 (Table 4). Of those receiving systemic antimonials either in monotherapy or in combination, 9.5% recorded an ADR, as did 51.1% of those receiving miltefosine.

**Table 4:** Adverse drug reactions in children.

	Systemic antimonials	Intralesional antimonials	Miltefosine	Total
**Total of ADRs, n**	**136**	**1**	**37**	**174**
**Total patients with ADRs, n** (% of patients receiving the treatment) *	63/663 (9.5%)	1/16 (6.3%)	22/43 (51.1%)	86/722 (11.9%)
**System organ class, n (%)**				
Cardiac	4 (2.9%)	0 (0%)	0 (0%)	4 (2.3%)
Gastrointestinal	32 (23.5%)	0 (0%)	26 (70.3%)	58 (33.3%)
General disorders and administration site conditions	29 (21.3%)	1 (100%)	1 (2.7%)	31 (17.8%)
Immune system	2 (1.5%)	0 (0%)	0 (0%)	2 (1.2%)
Investigations	4 (2.9%)	0 (0%)	0 (0%)	4 (2.3%)
Metabolism and nutrition	16 (11.8%)	0 (0%)	3 (8.1%)	19 (10.9%)
Musculoskeletal and connective tissue	13 (9.6%)	0 (0%)	0 (0%)	13 (7.5%)
Nervous system	25 (18.4%)	0 (0%)	6 (16.2%)	31 (17.8%)
Respiratory, thoracic and mediastinal	1 (0.7%)	0 (0%)	0 (0%)	1 (0.6%)
Skin and subcutaneous tissue	10 (7.4%)	0 (0%)	1 (2.7%)	11 (6.3%)
**Ten most frequent ADRs, regardless of the SOC (CTCAE): n (% of total ADRs)**				
Abdominal pain	7 (5.2%)	0 (0%)	2 (5.4%)	9 (5.2%)
Anorexia	15 (11.0%)	0 (0%)	3 (8.1%)	18 (10.3%)
Diarrhea	8 (5.9%)	0 (0%)	4 (10.8%)	12 (6.9%)
Dizziness	4 (2.9%)	0 (0%)	4 (10.8%)	8 (4.6%)
Fever	8 (5.9%)	0 (0%)	1 (2.7%)	9 (5.2%)
Headache	12 (8.8%)	0 (0%)	2 (5.4%)	14 (8.1)
Injection site reactions	8 (5.9%)	1 (100%)	0 (0%)	9 (5.2%)
Myalgia	8 (5.9%)	0 (0%)	0 (0%)	8 (4.6%)
Nausea	6 (4.4%)	0 (0%)	8 (21.6%)	14 (8.1%)
Vomiting	8 (5.9%)	0 (0%)	12 (32.4%)	20 (11.5%)
**Duration, days. Mean (SD) (n = 150)**	2.7 (3.6)	-	8.2 (9.3)	3.8 (5.7)
**Intensity (n = 163)**				
Mild: n (%)	116 (92.8%)	0 (0%)	35 (94.6%)	151 (92.6%)
Moderate: n (%)	7 (5.6%)	1 (100%)	2 (5.4%)	10 (6.1%)
Severe: n (%)	2 (1.6%)	0 (0%)	0 (0%)	2 (1.2%)
Life threatening	0 (0%)	0 (0%)	0 (0%)	0 (0%)
**Did the ADR require a concomitant treatment? (n = 78)**				
No: n (%)	23 (50%)	0 (0%)	25 (80.7%)	48 (61.5%)
Yes: n (%)	23 (50%)	1 (100%)	6 (19.3%)	30 (38.5%)
**Serious ADR: (n = 167)**				
No: n (%)	127 (98.5%)	1 (100%)	37 (100%)	165 (98.8%)
Yes: n (%)	2 (1.5%)	0 (0%)	0 (0%)	2 (1.2%)

*ADRs to systemic antimonials, whether a systemic antimonial was received alone or in combination. ADRs: Adverse drug reactions; SOC: System Organ Class; CTCTAE: Common Terminology Criteria for Adverse Events.

Overall, gastrointestinal disorders were the most frequently reported ADR group (33.3% of events). Nervous system disorders represented the second most reported group of ADRs (e.g., headache, somnolence) together with general disorders and administration site conditions (17.8%). Most of the ADRs were mild (92.6%, 151/163) and 6.1% were moderate. Mean ADR duration was 3.8 days (SD: 5.7). Two serious ADR were reported, both to systemic antimonials, one of which resolved completely, and the other having missing outcome data.

#### Adults ≥ 60 years of age

This patient group presented a higher frequency of ADRs to the main treatment regimens than children, as expected. In total, 206 adult patients (38.4%; 206/537) presented ADRs. In this group 536 ADRs were reported (Table 5). Among the most common regimens, 39.1% of those on systemic antimonials in monotherapy or in combination reported at least one related ADR, 34.5% of those receiving intralesional antimonials, 40.3% on liposomal amphotericin B, and 68.0% on miltefosine.

**Table 5:** Adverse drug reactions in adults ≥ 60 years old.

	Systemic antimonials	IL antimonials	Liposomal amphotericin B	Miltefosine	Pentamidine	Total
**Total of ADRs, n**	**401**	**52**	**34**	**45**	**4**	**536**
**Total patients with ADRs, n** (% of patients receiving the treatment) *	131/335 (39.1%)	29/84 (34.5%)	25/62 (40.3%)	17/25 (68.0%)	4/31 (12.9%)	206/537 (38.4%)
**ADRs per system organ class (CTCAE): n (%)**						
Blood and lymphatic system	0 (0%)	0 (0%)	2 (5.9%)	0 (0%)	0 (0%)	2 (0.4%)
Cardiac	23 (5.7%)	2 (3.9%)	0 (0%)	0 (0%)	0 (0%)	25 (4.6%)
Ear and Labyrinth	0 (0%)	1 (1.9%)	0 (0%)	0 (0%)	0 (0%)	1 (0.2%)
Gastrointestinal	51 (12.7%)	1 (1.9%)	0 (0%)	36 (80.0%)	2 (50%)	90 (16.7%)
General disorders and administration site conditions	66 (16.5%)	35 (67.3%)	1 (2.9%)	4 (8.9%)	0 (0%)	106 (19.8%)
Immune system	0 (0%)	1 (1.9%)	0 (0%)	0 (0%)	0 (0%)	1 (0.2%)
Infections and infestations	3 (0.8%)	1 (1.9%)	0 (0%)	0 (0%)	0 (0%)	4 (0.7%)
Investigations	29 (7.2%)	1 (1.9%)	18 (52.9%)	0 (0%)	0 (0%)	48 (9.0%)
Metabolism and nutrition	25 (6.2%)	0 (0%)	6 (17.7%)	2 (4.4%)	1 (25%)	34 (6.3%)
Musculoskeletal and connective tissue	99 (24.7%)	1 (1.9%)	1 (2.9%)	1 (2.2%)	1 (25%)	103 (19.1%)
Nervous system	75 (18.7%)	2 (3.9%)	1 (2.9%)	2 (4.4%)	0 (0%)	80 (14.8%)
Psychiatric	3 (0.8%)	0 (0%)	0 (0%)	0 (0%)	0 (0%)	3 (0.6%)
Renal and urinary	4 (1%)	0 (0%)	3 (8.8%)	0 (0%)	0 (0%)	7 (1.3%)
Respiratory, thoracic and mediastinal	0 (0%)	0 (0%)	1 (2.9%)	0 (0%)	0 (0%)	1 (0.2%)
Skin and subcutaneous tissue	22 (5.5%)	7 (13.5%)	0 (0%)	0 (0%)	0 (0%)	29 (5.4%)
Vascular	1 (0.3%)	0 (0%)	1 (2.9%)	0 (0%)	0 (0%)	2 (0.4%)
**Ten most frequent ADRs, regardless of the SOC (CTCAE): n (% of total ADRs)**						
Anorexia	17 (4.2%)	0 (0%)	0 (0%)	2 (4.4%)	0 (0%)	19 (3.5%)
Arthralgia	57 (14.2%)	0 (0%)	0 (0%)	1 (2.2%)	0 (0%)	58 (10.8%)
Increased creatinine	7 (1.8%)	1 (1.9%)	18 (52.9%)	0 (0%)	0 (0%)	26 (4.9%)
Fatigue	15 (3.7%)	0 (0%)	0 (0%)	1 (2.2%)	0 (0%)	16 (3.0%)
Headache	46 (11.5%)	0 (0%)	0 (0%)	2 (4.4%)	0 (0%)	48 (9.0%)
Injection site reactions	9 (2.2%)	34 (65.4%)	0 (0%)	0 (0%)	0 (0%)	43 (8.0%)
Malaise	21 (5.2%)	0 (0%)	1 (2.9%)	2 (4.4%)	0 (0%)	24 (4.5%)
Myalgia	38 (9.5%)	1 (1.9%)	0 (0%)	0 (0%)	1 (25%)	40 (7.5%)
Nausea	27 (6.7%)	0 (0%)	0 (0%)	12 (26.7%)	1 (25%)	40 (7.5%)
Serum amylase increased	18 (4.5%)	0 (0%)	0 (0%)	0 (0%)	0 (0%)	18 (3.4%)
**Duration, days. Mean (SD) (n = 411)**	4.6 (11.9)	3.7 (4.3)	11 (13.3)	9.8 (11.8)	1 (0)	5.1 (11.7)
**Intensity (n = 469)**						
Mild: n (%)	277 (76.5%)	25 (86.2%)	23 (79.3%)	37 (82.2%)	4 (100%)	366 (78.0%)
Moderate: n (%)	81 (22.4%)	3 (10.3%)	5 (17.2%)	7 (15.6%)	0 (0%)	96 (20.5%)
Severe: n (%)	4 (1%)	1 (3.5%)	1 (3.5%)	1 (2.2%)	0 (0%)	7 (1.5%)
Life threatening: n (%)	0 (0%)	0 (0%)	0 (0%)	0 (0%)	0 (0%)	0 (0%)
**Did the ADR require a concomitant treatment? (n = 265)**						
No: n (%)	65 (39.6%)	28 (66.7%)	26 (83.9%)	13 (54.2%)	3 (75%)	135 (50.9%)
Yes: n (%)	99 (60.4%)	14 (33.3%)	5 (16.1%)	11 (45.8%)	1 (25%)	130 (49.1%)
**Serious ADR: (n = 485)**						
No: n (%)	356 (97.3%)	37 (94.9%)	28 (90.3%)	45 (100%)	4 (100%)	470 (96.9%)
Yes: n (%)	10 (2.7%)	2 (5.1%)	3 (9.7%)	0 (0%)	0 (0%)	15 (3.1%)

*ADRs to systemic antimonials, whether a systemic antimonial was received alone or in combination

ADRs: Adverse drug reactions; SOC: System Organ Class; CTCTAE: Common Terminology Criteria for Adverse Events

For antimonials, the system or organs most affected were mainly those already described in the literature [17], with a predominance of musculoskeletal and connective tissue manifestations (n = 99; 24.7% of events), followed by nervous system symptoms (n = 75; 18.7%). By contrast, the ADRs for liposomal amphotericin B were mostly laboratory result abnormalities (n = 18, 52.9%) such as increases of creatinine, hypokalemia, and other electrolyte imbalances. Most of the ADRs were of mild intensity (78.0%; 366/469) and 20.5% (n = 96/469) were moderate. Average ADR duration was 5.1 days (SD: 11.7). Fifteen serious ADRs were reported (10 to systemic, two to IL antimonials and three to amphotericin B), of which 11 resolved completely, two were continuing at end of follow-up, and two had missing outcome data.

## Discussion

This multi-institution collaborative study provides evidence of the effectiveness of antileishmanial treatments in special populations (children ≤ 10 years of age and adults ≥ 60 years of age) in Latin America. This study of 1,325 CL patients from ten participating sites in four countries is, to our knowledge, one of the largest such studies in the region. The population within the eligible age groups screened for this study represented 21.4% of the patients attending the participating referral centers during the corresponding 5-year period, a proportion similar to that reported for these age groups in the Americas [3].

The limited follow-up data for the different antileishmanial treatments remains a challenge in the region. This was evident during the screening process. Of the 242 and 468 found to be ineligible in children and older adults respectively, approximately 75% and 40% were due to the lack of data on treatment follow-up and therapeutic response. Furthermore, the proportion of children with more than one follow-up visit was also lower than in adults, with only 28% of them having data for two follow-up visits versus 48.7% of patients ≥ 60 years. National treatment guidelines often recommend three or more follow-up visits after completion of treatment [7,18–20]. However, logistic and economic challenges restrict access to treatment follow-up, especially for these more vulnerable age groups, as some of these patients need to be accompanied by parents or guardians. Our findings align with reports from PAHO, in which 38.5% of cases have no information on treatment outcomes in several countries and the region overall [3], and underscore the need to develop strategies to improve the monitoring of treatment response and effectiveness of the standard of care in the region, with special attention to pediatric patients.

Disease presentation was mild, with the median number of lesions being one and the average size of lesion < 32 mm for both children and older adults. The mild clinical presentation of disease in both study groups make them potentially eligible for local therapies such as intralesional antimonials or thermotherapy, as per PAHO treatment recommendation guidelines [6,21]. Revised PAHO guidelines published in 2022 consolidate and expand the use of local interventions for patients with localized CL with a maximum of three lesions of no more than three cm in diameter [21]. Eligibility for local therapies is contraindicated by the presence of lesions on head and neck, which is more frequent in children [22]. However, local interventions were only used in a small number of patients in the study cohort, e.g., thermotherapy was used in only 21 (1.6%) patients in the entire cohort—19 adults and 2 children —far less than the estimated eligible proportion reported [22, 23].

Overall compliance with drug dosing schemes recommended by PAHO guidelines [6] was generally high, ranging from more than 90% for antimonials to approximately 70% for miltefosine and liposomal amphotericin B in adults. Notably, two of the sites used dosing regimens in the lower range of the recommendations: meglumine antimoniate dosage of 5 mg Sb^V+^/kg/day for 30 days is used routinely at INI [24–26] and lower doses of liposomal amphotericin B in Corte de Pedra/C-HUPES.

The overall clinical cure in response to systemic antimonials in children was low (54.6%; 95% CI: 50.46–58.6%), as previously reported in the few clinical trials including children [10,27] and in a cohort study that showed increased risk of failure in children ≤ 8 years of age [28]. The lower effectiveness of pentavalent antimonials is partially explained by pharmacokinetic differences of the drug in children, who have an increased elimination rate compared to adults [29]. The geographic location of treatment did not seem to influence the overall cure for systemic antimonials, which reached 64.2% with overlapping 95% CI (104/162; 95% CI: 56.3–71.6%) when excluding the patients from CSCP (Fig 2A), the site that contributed the most pediatric cases and where *L*. *(V*.*) braziliensis* predominates. Overall cure rate for miltefosine, the second most frequently used monotherapy in the study population, was similar to that for antimonials (55.8%; 95% CI: 39.8%-70.9%), though lower than reported in clinical trials that included children conducted in the region, which ranged from 63.3% to 87.3% [10]. However, the number of children treated with miltefosine in our study was small (n = 43) representing only 5.8% of the study population. Results of pharmacokinetics studies of miltefosine in children have stressed the need to allometrically adapt the dose in pediatric populations to improve efficacy [30–34].

In adults ≥ 60 years of age, the overall cure rate for systemic antimonials was higher than in children (68.2%; 95% CI: 62.6–73.39%) and in line with rates reported in clinical trials for systemic antimonials in the region, ranging from less than 50% [35, 36]to more than 90% [37–40]. A smaller retrospective study conducted at Corte de Pedra reported a similar cure rate for systemic antimonials in this population ≥60 years of age [41]. The loss of efficacy of systemic antimonials for the treatment of CL due to *L*. *braziliensis* in the endemic area of Corte de Pedra (Brazil) has been documented, with a cure rate of 53% [42,43]. Patients from this site accounted for 33% of older adults, however, the overall cure rate in response to systemic antimonials when excluding the patients from CSCP remained close to the estimate in the whole cohort (70%; 95% CI: 62.0–77.2%), Fig 2B.

The second most frequently used therapy in this group was intralesional antimonials, which in our study presented an overall cure rate of 84.5% (95% CI: 74.99–91.5%). This is higher than in a smaller retrospective study in Brazil, which reported a 73.3% cure rate [44] but still aligned with the overall efficacy of intralesional antimonials of 76.9% (95% CI: 66–85%), reported in a literature review for American CL. This review reported a somewhat higher efficacy of 84.6% (95% CI 72.2–92.1%) in non-randomized studies [45]. Notably, median lesion size (13mm; IQR: 6–25) in patients treated with intralesional antimonials was smaller than in patients receiving other therapies such as systemic antimonials (26mm; IQR: 15–45) or miltefosine (22mm; IQR: 13–35), S7 Table.

In terms of the reported adverse drug reactions, the type, and the system or organs affected for each therapeutic intervention are mainly those described in clinical trials populations, of mild intensity and expected from medication package inserts. As expected, safety data showed a higher frequency of patients presenting ADRs in older adults than in children. The proportion of children with ADRs in this study is lower than the 50% described in another retrospective study with a pediatric cohort treated with systemic antimonials [46]. Serious adverse drug reactions in children were rare. As previously described [10,46], among those with ADRs, children were more likely to present mild ADRs than older adults (92.6% versus 78.0% in the current study) reflecting the higher elimination rate in children and co-morbidities in older adults. The proportion of patients on miltefosine with ADRs was respectively 51.1% in children and 68.9% in older adults, similar to that previously reported [30]. No serious ADRs to miltefosine were reported, while most of the reported serious ADRs were related to systemic antimonials. This magnitude of ADRs related to miltefosine might also be explained by the fact that some patients, mostly children, were treated with miltefosine in the framework of an observational study where systematic reporting of ADRs occurred. However, in our study, ADRs related to other treatments were not always systematically elicited for clinical records, and hence their frequency may have been underestimated. This heterogeneity in elicitation of ADRs is a limitation of our study.

Besides potential issues inherent to retrospective studies in terms of quality of registered data, another limitation of this study is the scarcity of data on *Leishmania* species identification, which was only available for less than 10% of participants and hindered our ability to make inferences on treatment effectiveness according to the species. This may be due to the cost, and logistic and technical requirements for isolation and identification of the *Leishmania* species. Also, since a low proportion of patients had data on therapeutic response between D90 and D100 (mostly for systemic antimonials), evaluation of initial cure was limited. Considering the high proportion of patients coming from one site (50% from Corte de Pedra) as a potential source of bias, we performed a sensitivity analysis of overall cure rate excluding the patients treated in that site, which did not indicate an impact of the site on the overall cure rate for systemic antimonials. Losses to follow-up may affect our measures of treatment effectiveness, which might have been under-estimated because cured patients possibly return with less frequency for follow-up to health services, as most of them live in remote rural areas and returning to the clinic is costly and time-consuming. Other factors unrelated to treatment outcome, such as migration of seasonal coffee workers [47], may also contribute to losses to follow-up. Therefore, improving patient retention is key to ensure accurate measures of treatment effectiveness in the region.

One of the strengths of the study is the generalizability of our results. Brazil, Colombia, Peru, and Bolivia are the countries reporting most CL cases in the Americas, and together with Nicaragua represented 81% of all cases reported from the region in 2020 [2]. The ten study sites are reference centers for CL in these countries, treating a proportion of pediatric cases similar to that previously reported in the region (S2 Table) [2], and the demographic and clinical characteristics of our study participants were aligned with those described in studies of CL in the region [22,41,46,48–51].

Our findings on the predominance of mild disease presentation and limited effectiveness of systemic antimonials in pediatric and older adult patients support increasing the adoption of and access to alternative treatment options for these special populations. The proportion of children treated with systemic antimonials was 80.2%, despite the evidence of lower efficacy that motivated the change in PAHO guidelines, published in 2022, which now strongly recommend the use of miltefosine for children, leaving systemic antimonials only when no other alternatives are available [21]. The use of systemic antimonials for older adults was also high (52%), despite this treatment being only conditionally recommended for adults aged older than 50 years [21]. As mentioned above, use of local therapies in this study was far less than the proportion of potentially eligible patients reported in similar populations with mild CL [22,23]. Increasing access to local therapies will contribute to the achievement of critical action 1 for CL of the WHO-NTD 2030 roadmap, “Develop and scale up easy-to administer oral or topical treatments that could be used in health centers” [52]. This involves their inclusion in national treatment recommendation guidelines, availability of standardized protocols and equipment (e.g., for thermotherapy), and training of healthcare providers in the application of these interventions.

Other therapeutic options that need to be made increasingly available include miltefosine and liposomal amphotericin B. The latter is already recommended as a first line option in patients of ≥ 50 years of age in Brazil [7] and for children weighing less than 10 kg in Colombia [18], but its high cost and supply constraints limit access and use. When analyzing the prescription of drugs other than systemic antimonials, the reporting country was an important factor. For example, all patients treated with liposomal amphotericin B were from Brazil and 84% of patients treated with miltefosine were from Colombia (S7 Table). During the period of the study, and in the countries where the participating centers are located, miltefosine was only available in Colombia and was recommended as first line treatment for children above 10 kg [18]. Since then, it has been incorporated into the Brazilian Unified Health System as first line treatment for CL for patients of 12 years of age and above, with implementation initiated in 2021 [53,54] Moreover, the revised PAHO guidelines give miltefosine a strong recommendation for the treatment of CL patients with lesions caused by *L*. *panamensis*, *L*. *guyanensis*, *L*. *mexicana* and *L*. *braziliensis*, and this includes the pediatric population [21]. However, its presentation in capsule form is a challenge for administration in younger children, underscoring the need for the development of new pediatric formulations. Finally, strategies for improving patient follow-up and data on treatment effectiveness are needed to guide decisions and policy. Standardization of data collection formats and strategies regarding treatment follow-up, strengthening of health surveillance, telemedicine, mHealth tools, and other measures for active case follow-up would contribute to increased and more complete information on treatment outcomes and ultimately improve the care of these special populations. Standardization of data collection formats would also promote regional data sharing.

This collaborative study presents real-world evidence of the use and effectiveness of antileishmanial drugs in special populations (children and older adults) in Latin America. The methodology for recovery, management and sharing of data from diverse centers providing clinical attention to an NTD is a new approach and proof of concept. It was necessary to develop instruments to collect, process, analyze and interpret data that were otherwise unavailable or fragmented within the region. Our findings from ten reference centers in the countries with the highest burden of CL in Latin America support the need for wider implementation of local therapies, availability of alternatives to systemic antimonials, and development of strategies to improve patient follow-up across the region.

## Supporting information

S1 Table STROBE checklist [11]. (DOC)

S2 Table Distribution of patients attended and included per country and study site during the study period. (DOCX)

S3 Table Diagnostic methods per study group. (DOCX)

S4 Table Therapeutic response in children. (DOCX)

S5 Table Therapeutic response in adults ≥ 60 years old. (DOCX)

S6 Table Treatment regimens and adherence to PAHO dose recommendations. (DOCX)

S7 Table Monotherapy treatment regimens in patients ≥60 years old by country and clinical characteristics. (DOCX)
