# Supplementary material for: Cutaneous leishmaniasis treatment and therapeutic outcomes in special populations: A collaborative retrospective study
Source: PLoS Negl Trop Dis. 2023 Jan 23;17(1):e0011029. doi: 10.1371/journal.pntd.0011029 (PMC9894540; doi:10.1371/journal.pntd.0011029)
Supplement: S2 Table — (DOCX) [file pntd.0011029.s002.docx]

**S2 Table.** Distribution of patients attended and included per country and study site during the study period

| *Country /* Site | Total number of cases attended | Number (%) cases ≤ 10 years attended | Number (%) cases ≥ 60 years attended | Number (%) cases > 10 years and < 60 years attended | Number of cases (%) ≤ 10 years included in the study | Number of cases (%) ≥ 60 years included in the study | Total number of cases (%) included in the study | |
| --- | --- | --- | --- | --- | --- | --- | --- | --- |
| *Bolivia* | | | | | | | | |
| CUMT | 330 | 36 (10.9%) | 27 (8.2%) | 267 (80.9%) | 36 (4.9%) | 12 (2%) | 48 (3.6%) | |
| Funderma | 426 | 7 (1.6%) | 37 (8.7%) | 382 (89.7%) | 2 (0.3%) | 9 (1.5%) | 11 (0.8%) | |
| *Brasil* | | | | | | | |  |
| CPqRR | 586 | 36 (6.1%) | 135 (23%) | 415 (70.8%) | 20 (2.7%) | 90 (15.3%) | 110 (8.3%) | |
| HUJM | 732 | 32 (4.4%) | 118 (16.1%) | 582 (79.5%) | 30 (4.1%) | 98 (16.6%) | 128 (9.7%) | |
| INI | 119 | 0 (0%) | 39 (32.8%) | 80 (67.2%) | 0 (0%) | 14 (2.4%) | 14 (1.1%) | |
| CSCP | 4.399 | 504 (11.5%) | 401 (9.1%) | 3494 (79.4%) | 427 (58%) | 194 (32.9%) | 621 (46.9%) | |
| *Colombia* | | | | | | | |  |
| CDFLA | 230 | 49 (21.3%) | 49 (21.3%) | 132 (57.4%) | 41 (5.6%) | 36 (6.1%) | 77 (5.8%) | |
| CIDEIM | 966 | 108 (11.2%) | 41 (4.2%) | 817 (84.6%) | 45 (6.1%) | 16 (2.7%) | 61 (4.6%) | |
| PECET | 593 | 53 (8.9%) | 51 (8.6%) | 489 (82.5%) | 20 (2.7%) | 20 (3.4%) | 40 (3%) | |
| *Peru* | | | | | | | |  |
| UPCH | 1284 | 185 (14.4%) | 161 (12.5%) | 938 (73.1%) | 115 (15.6%) | 100 (17%) | 215 (16.2%) | |
| Total | 9665 | 1010 (10.5%) | 1059 (11%) | 7596 (78.6%) | 736 (55.5%) | 589 (44.5%) | 1325 (100%) | |
