# Supplementary material for: Cutaneous leishmaniasis treatment and therapeutic outcomes in special populations: A collaborative retrospective study
Source: PLoS Negl Trop Dis. 2023 Jan 23;17(1):e0011029. doi: 10.1371/journal.pntd.0011029 (PMC9894540; doi:10.1371/journal.pntd.0011029)
Supplement: S3 Table — (DOCX) [file pntd.0011029.s003.docx]

**S3 Table.** Diagnostic methods per study group

|  | Patients ≤ 10 years | | Patients ≥ 60 years | |
| --- | --- | --- | --- | --- |
| Total number of cases | 736 | | 589 | |
| **Type of diagnosis: n (%)** | **736** | **(100%)** | **589** | **(100%)** |
| Clinical-epidemiological | 467 | (63.5%) | 149 | (25.3%) |
| Parasitological | 269 | (36.5%) | 440 | (74.7%) |
| **In patients with parasitological diagnosis, tests performed: n (% of total)** |  |  |  |  |
| **Direct smear** | 269 | (100%) | 334 | (75.9%) |
| Positive | 246 | (91.4%) | 279 | (83.5%) |
| Negative | 21 | (7.8%) | 41 | (12.3%) |
| No data | 2 | (0.7%) | 14 | (4.2%) |
| **Culture** | 106 | (39.4%) | 238 | (54.1%) |
| Positive | 39 | (36.8%) | 118 | (49.6%) |
| Negative | 16 | (15.1%) | 51 | (21.4%) |
| No data | 51 | (48.1%) | 69 | (29%) |
| **PCR** | 102 | (37.9%) | 243 | (55.2%) |
| Positive | 13 | (12.7%) | 121 | (49.8%) |
| Negative | 4 | (3.9%) | 6 | (2.5%) |
| No data | 85 | (83.3%) | 116 | (47.7%) |
| **Immunohistochemistry** | 59 | (21.9%) | 140 | (31.8%) |
| Positive | 0 | (0%) | 9 | (6.4%) |
| Negative | 2 | (3.4%) | 7 | (5%) |
| No data | 57 | (96.6%) | 124 | (88.6%) |
| **Histopathology** | 107 | (39.8%) | 255 | (58%) |
| Positive | 8 | (7.5%) | 40 | (15.7%) |
| Negative | 45 | (42.1%) | 5 | (2%) |
| Suggestive | 8 | (7.5%) | 39 | (15.3%) |
| No data | 46 | (43%) | 171 | (67.1%) |
| **Positive cases according to test: n (% with test)** |  |  |  |  |
| Direct Smear | 246 | (92.1%) | 279 | (87.2%) |
| Culture | 39 | (70.9%) | 118 | (69.8%) |
| PCR | 13 | (76.5%) | 121 | (95.3%) |
| Immunohistochemistry | 0 | (0%) | 9 | (56.3%) |
| Histopathology | 8 | (13.1%) | 40 | (47.6%) |
| ***Leishmania* species: n (% of number with identified species)** | **17** | **(6.3%)** | **107** | **(24.3%)** |
| *Peruviana* | 0 | (0%) | 0 | (0%) |
| *Braziliensis* | 0 | (0%) | 95 | (88.8%) |
| *Guyanensis* | 1 | (5.9%) | 0 | (0%) |
| *Panamensis* | 15 | (88.2%) | 11 | (10.3%) |
| *Amazonensis* | 0 | (0%) | 0 | (0%) |
| *Mexicana* | 1 | (5.9%) | 0 | (0%) |
| *L. infantum* | 0 | (0%) | 1 | (0.9%) |
