# Supplementary material for: Cutaneous leishmaniasis treatment and therapeutic outcomes in special populations: A collaborative retrospective study
Source: PLoS Negl Trop Dis. 2023 Jan 23;17(1):e0011029. doi: 10.1371/journal.pntd.0011029 (PMC9894540; doi:10.1371/journal.pntd.0011029)
Supplement: S4 Table — (DOCX) [file pntd.0011029.s004.docx]

**S4 Table.** Therapeutic response in children.

|  | Systemic antimonials | IL antimonials | Miltefosine | Systemic antimonials +imiquimod |
| --- | --- | --- | --- | --- |
| Total | 590 | 16 | 43 | 66 |
| **Number of cases with evaluation at days 42-90** | **189** | **5** | **17** | **31** |
| Cure: n (%) | 104 (55%) | 2 (40%) | 11 (64.7%) | 16 (51.6%) |
| 95% confidence interval | (47.6%- 62.3%) | (5.2%- 85.3%) | (38.3%- 85.7%) | (33.06%- 69.8%) |
| ***Initial cure*** | | | | |
| **Number of cases with evaluation at days 90-100** | 196 | 2 | 6 | 4 |
| Cure: n (%) | 102 (52%) | 1 (50%) | 3 (50%) | 1 (25%) |
| 95% confidence interval | (44.8%-59.2%) | (1.2%-98.7%) | (11.8%-88.1%) | (0.6%-80.5%) |
| ***Overall analysis (cumulative cases)*** | | | | |
| ***Overall cure*** | | | | |
| **Number of cases** | 588 | 16 | 43 | 65 |
| Cure: n (%) | 321 (54.6%) | 10 (62.5%) | 24 (55.8%) | 29 (44.6%) |
| 95% confidence interval | (50.46%-58.6%) | (35.4%-84.8%) | (39.8%-70.9%) | (32.26%-57.4%) |
| ***Therapeutic failure*** | | | | |
| Failure: n (%) | 232 (39.5%) | 4 (25%) | 10 (23.3%) | 22 (33.8%) |
| 95% confidence interval | (35.4%-43.5%) | (7.2%-52.3%) | (11.7%-38.6%) | (22.5%-46.6%) |
| ***Relapse*** | | | | |
| **Number of cases with data about relapse: *n*** | 325 | 1 | 5 | 12 |
| Relapse: n (%) | 37 (11.4%) | 1 (100%) | 0 (0%) | 7 (58.3%) |
| 95% confidence interval | (8.1%-15.3%) | (2.5%-100%^h^) | (0%-52.1%^h^) | (27.6%-84.8%) |

^h^ (*) one-sided, 97.5% confidence interval
