# Supplementary material for: Cutaneous leishmaniasis treatment and therapeutic outcomes in special populations: A collaborative retrospective study
Source: PLoS Negl Trop Dis. 2023 Jan 23;17(1):e0011029. doi: 10.1371/journal.pntd.0011029 (PMC9894540; doi:10.1371/journal.pntd.0011029)
Supplement: S5 Table — (DOCX) [file pntd.0011029.s005.docx]

S5 Table. Therapeutic response in adults ≥ 60 years old

|  | Systemic antimonials | IL antimonials | Amphotericin B liposomal | Miltefosine | Pentamidine | Other treatments (monotherapy) | Systemic antimonial+ pentoxifylline | Other combinations |
| --- | --- | --- | --- | --- | --- | --- | --- | --- |
| Total | 308 | 84 | 62 | 25 | 31 | 35 | 19 | 24 |
| **Number of cases with evaluation at days 42-90** | 136 | 27 | 40 | 9 | 13 | 14 | 13 | 16 |
| Cure: n (%) | 101 (74.3%) | 23 (85.2%) | 18 (45%) | 6 (66.7%) | 9 (69.2%) | 6 (42.9%) | 12 (92.3%) | 12 (75%) |
| 95% confidence interval | (66.1 -81.4%) | (66.3 -95.8%) | (29.3 -61.5%) | (29.9 -92.5%) | (38.6 -90.9%) | (17.7 -71.1%) | (63.9 - 99.8%) | (47.6 - 92.7%) |
| ***Initial cure*** | | | | | | | | |
| **Number of cases with evaluation at days 90-100** | 61 | 23 | 8 | 5 | 4 | 2 | 1 | 2 |
| Cure: n (%) | 45 (73.8%) | 18 (78.3%) | 7 (87.5%) | 4 (80%) | 0 (0%) | 1 (50%) | 1 (100%) | 2 (100%) |
| 95% confidence interval | (60.9 -84.2%) | (56.3 -92.5%) | (47.3 -99.7%) | (28.4 -99.5%) | (0 -60.24%^h^) | (1.26 -98.74%) | (2.5 -100%^h^) | (15.81 -100%^h^) |
| ***Overall analysis (cumulative cases)*** | | | | | | | | |
| ***Overall cure*** | | | | | | | | |
| **Number of cases** | 305 | 84 | 62 | 25 | 31 | 32 | 19 | 24 |
| Cure: n (%) | 208 (68.2%) | 71 (84.5%) | 31 (50%) | 14 (56%) | 17 (54.8%) | 13 (40.6%) | 16 (84.2%) | 16 (66.7%) |
| 95% confidence interval | (62.6 -73.39%) | (74.99 -  91.5%) | (37.02 -  62.97%) | (34.9 -  75.6%) | (36.0 -  72.7%) | (23.7 -  59.4%) | (60.4 -  96.6%) | (44.7 -  84.4%) |
| ***Therapeutic failure*** | | | | | | | | |
| Failure: n (%) | 64 (21%) | 10 (11.9%) | 13 (21%) | 6 (24%) | 5 (16.1%) | 14 (43.8%) | 1 (5.3%) | 5 (20.8%) |
| 95% confidence interval | (16.6 -25.98%) | (5.86 -20.8%) | (11.7 -33.2%) | (9.4 - 45.1%) | (5.5 - 33.7%) | (26.4 -62.3%) | (1.3 - 26.0%) | (7.1 - 42.2%) |
| ***Relapse*** | | | | | | | | |
| **Number of cases with data about relapse: *n*** | 172 | 32 | 28 | 6 | 7 | 9 | 3 | 6 |
| Relapse: n (%) | 22 (12.8%) | 2 (6.3%) | 4 (14.3%) | 2 (33.3%) | 1 (14.3%) | 0 (0%) | 1 (33.3%) | 2 (33.3%) |
| 95% confidence interval | (8.2 -18.7%) | (0.77 -20.8%) | (4.0 -32.7%) | (4.3 -77.7%) | (0.36 -57.9%) | (0 -33.6%^h^) | (0.84 -90.6%) | (4.3 -77.7%) |

^h^ (*) one-sided, 97.5% confidence interval
