# Supplementary material for: Cutaneous leishmaniasis treatment and therapeutic outcomes in special populations: A collaborative retrospective study
Source: PLoS Negl Trop Dis. 2023 Jan 23;17(1):e0011029. doi: 10.1371/journal.pntd.0011029 (PMC9894540; doi:10.1371/journal.pntd.0011029)
Supplement: S6 Table — (DOCX) [file pntd.0011029.s006.docx]

S6 Table. Treatment regimens and adherence to PAHO dose recommendations.

|  | Systemic antimonials | | IL antimonials | | Amphotericin B (liposomal) | | Miltefosine | | Pentamidine^a^ | | | |
| --- | --- | --- | --- | --- | --- | --- | --- | --- | --- | --- | --- | --- |
|  | 10 – 20mg/kg/day | | 81-405 mg per lesion/session | | 2-3 mg/kg/day | | 1.5-2.5 mg/kg/day | | 3-4 mg/kg/day | |  |  |
| *Children* | 663 | | 16 | | 5 | | 43 | | 3 | | | |
| Dose within the recommended range: n (%) | 649 | (97.9%) | 15 | (93.8%) | 5 | (100%) | 34 | (79.1%) | 1 | (33.3%) | | |
| Among these, Mean dose (SD) | 19.1 | (1.78) | 208.76 | (176.23) | 2.5 | (0.5) | 2.3 | (0.2) | 4 | - | | |
| Dose lower than recommended, Mean (SD) | 9.52 | - | - | - | - | - | 1.2 | (0.22) | - | - | | |
| Dose higher than recommended, Mean (SD) | 22.05 | (1.6) | - | - | - | - | 2.7 | (0.1) | - | - | | |
| *Adults* ≥60 years | 335 | | 87 | | 62 | | 32 | | 31 | | |  |
| Dose within the recommended range: n (%) | 321 | (95.8%) | 81 | (93.1%) | 43 | (69.4%) | 25 | (78.1%) | 25 | (80.6%) | | |
| Among these, Mean dose (SD) | 17.06 | (3.27) | 512.9 | (972.7) | 2.7 | (0.46) | 2.23 | (0.25) | 3.59 | (0.44) | | |
| Dose lower than recommended, Mean (SD) | 6.51 | (1.91) | - | - | 1.6 | (0.19) | 0.94 | - | - | - | | |
| Dose higher than recommended, Mean (SD) | 21.5 | - | - | - | - | - | 3.01 | (0.22) | 5.79 | (0.71) | | |

Notes a) All doses are presented in mg/kg/day, except intralesional (IL) antimonials, which are presented as mg/day. b) Cases who received each drug are included, whether or not that drug was received as monotherapy or in combination.
