# Supplementary material for: Cutaneous leishmaniasis treatment and therapeutic outcomes in special populations: A collaborative retrospective study
Source: PLoS Negl Trop Dis. 2023 Jan 23;17(1):e0011029. doi: 10.1371/journal.pntd.0011029 (PMC9894540; doi:10.1371/journal.pntd.0011029)
Supplement: S7 Table — (DOCX) [file pntd.0011029.s007.docx]

S7 Table. Monotherapy treatment regimens in patients ≥60 years old by country and clinical characteristics

|  | Systemic antimonials | IL antimonials | Amphotericin B (liposomal) | Miltefosine | Pentamidine |
| --- | --- | --- | --- | --- | --- |
| Number of adults ≥60 years | 308 | 84 | 62 | 25 | 31 |
| Time from beginning of symptoms. Weeks. Median (IQR) | 8 (4-12) | 16 (8-20) | 8 (4-12) | 12 (8-32) | 12 (8-20) |
| Country n (%) |  |  |  |  |  |
| Colombia | 19 (6) | 15 (18) | 0 (0) | 21 (84) | 10 (32) |
| Brazil | 225 (73) | 67 (80) | 62 (100) | 3 (12) | 21 (68) |
| Peru | 46 (15) | 0 (0) | 0 (0) | 0 (0) | 0 (0) |
| Bolivia | 18 (6) | 2 (2) | 0 (0) | 1 (4) | 0 (0) |
| Any concomitant disease. n (%) | 163 (54) | 69 (87) | 50 (82) | 16 (64) | 19 (63) |
| Number of lesions. Median (IQR) | 1 (1-2) | 1 (1-1) | 1 (1-2) | 2 (1-4) | 1 (1-2) |
| Lesion size. Median (IQR) | 26 (15-45) | 13 (6-25) | 25 (12.5-40) | 22 (13-35) | 30 (25-40) |

IQR= interquartile range
